# Interpretation of Tumor Response Grade following Preoperative Therapy for Gastric Cancer: An Overview

**DOI:** 10.3390/cancers15143662

**Published:** 2023-07-18

**Authors:** Vasileios Tsagkalidis, Maryjka B. Blaszczyk, Haejin In

**Affiliations:** 1 Division of Surgical Oncology, Rutgers Cancer Institute of New Jersey, New Brunswick, NJ 08901, USA; vt308@cinj.rutgers.edu; 2 Department of Pathology and Laboratory Medicine, Rutgers Biomedical and Health Sciences, New Brunswick, NJ 08901, USA; mb2001@rwjms.rutgers.edu; 3 Rutgers Robert Wood Johnson Medical School, New Brunswick, NJ 08901, USA; 4 Department of Health Behavior, Society and Policy, Rutgers School of Public Health, Piscataway, NJ 08854, USA

**Keywords:** tumor response grade (TRG), gastric cancer, RECIST, tumor regression score

## Abstract

**Simple Summary:**

The tumor response grade (TRG) of gastric cancer following neoadjuvant therapy is used to quantify therapeutic response. Multiple grading systems exist, with different cut-off values and variable reproducibility. Studies have attempted to associate TRG with survival to assist with prognostication, with inconsistent results, likely secondary to the use of different grading systems between studies and interobserver variability. Radiographic and endoscopic response have also been evaluated as markers of treatment response, but their use in gastric cancer is challenging. This review provides an overview of the current literature regarding the use of TRG in gastric cancer, discussing the interpretation of radiographic and endoscopic response following preoperative treatment, as well as outlining future directions regarding aims to develop, implement, and validate a widely accepted TRG system.

**Abstract:**

Gastric cancer is among the top five causes of cancer-related death worldwide. Preoperative chemotherapy has been established as an option in patients with locally advanced gastric cancer. However, chemotherapy yields variable results, owing to the cellular and molecular heterogeneity of this disease. Identifying patients who did or did not respond to preoperative therapy can allow clinicians to alter treatment modalities and provide important information related to prognostication. A pathologic response to preoperative therapies, called the Tumor Response Grade (TRG), has been evaluated to quantify treatment response. Multiple systems for TRG have been established. However, the literature has demonstrated inconsistent results for TRG systems and prognosis, possibly due to variability in interpretation of tumor response between systems and interobserver variability. Radiographic responses to preoperative therapies using RECIST 1.1 criteria and endoscopically assessed tumor response have demonstrated association with survival; however, their use in gastric cancer remains challenging given the inability to accurately and consistently identify and measure the tumor, especially in the setting of neoadjuvant therapy, where treatment-related changes can obscure the gastric wall layers. While the response to preoperative therapies with positron emission tomography (PET) has shown promising results in esophageal and esophagogastric junction (EGJ) malignancies, its role in gastric cancer is still under investigation. This review is focused on summarizing the available literature related to evaluating TRG in gastric cancer, as well as providing a brief overview of the use of radiographic and endoscopic methods to assess response to preoperative therapies. Lastly, we outline future directions regarding the use of a universal TRG system to guide care and assist with prognosis.

## 1. Introduction

Gastric cancer is the fifth most common cancer worldwide and fourth most common cause of cancer-related death, with over 1 million new cases and nearly 800 thousand deaths reported in 2020 (GLOBOCAN, 2020) [1]. Neoadjuvant therapies, including chemotherapy, chemoradiation and immunotherapy, are part of the treatment armamentarium for gastric cancer. Retrospective studies demonstrate that neoadjuvant therapy is associated with improvement in survival, particularly for more advanced stages [2]. Preoperative therapies for select patients can provide a plethora of advantages compared to adjuvant treatments. They allow for possible downstaging, decreasing the size of the primary tumor, reducing the extent of lymph node involvement, treating micrometastatic disease, and subsequently improving survival by increasing the chances of complete resection and cure. Additionally, preoperative systemic therapy ensures the delivery of systemic therapy in patients, while therapy in the adjuvant setting may be hindered due to post-operative complications. Selecting the optimal preoperative treatment option depends on multiple factors, such as the histology, tumor stage, gene expression status, patient comorbidities, performance status and toxicity profile of each therapeutic agent. Assessing the response to preoperative chemotherapy can provide information regarding prognostication and help clinicians guide treatment in this patient population [3,4].

## 2. Gastric Cancer and Preoperative Therapies

Patients with gastric adenocarcinoma clinical T2 stage (invasion of the muscularis propria) or higher, irrespective of nodal status, are candidates for either upfront surgery followed by adjuvant chemoradiation, perioperative chemotherapy or upfront chemoradiation followed by surgery. In the US, chemotherapy with or without radiation prior to surgery is generally considered the standard of care for tumors T2 and higher. Upon completion of any preoperative systemic therapy, patients are restaged and then undergo surgical resection, if their disease remains resectable. The pathologic results of the surgical specimen provides an opportunity to reassess the choice of adjuvant treatment regimens to incorporate factors such as tumor response to treatment, completeness of surgical resection and pathologic assessment of tumor biology [3]. The Medical Research Council Adjuvant Gastric Infusional Chemotherapy (MAGIC) trial from the UK showed that patients with resectable clinical ≥ stage II (M0) disease treated with perioperative chemotherapy (three cycles pre- and three cycles post-operatively of epirubicin, cisplatin and 5FU [ECF]) had an improved 5-year survival rate of 36% versus 23% in the surgery-only group [5]. The FNCLCC/FFCD phase 3 trial from France showed similar results to the MAGIC trial, with patients who were treated with cisplatin-5FU followed by surgery having a 5-year survival rate of 38% compared to 24% in the surgery-only group [6]. In 2019, the results of a phase 2/3 trial from Germany showed that patients with >T2 and/or N-positive disease who received fluorouracil plus leucovorin, oxaliplatin and docetaxel (FLOT), had improved median overall survival (OS) to 50 months compared to 35 months in patients who received ECF/epirubicin–cisplatin–capecitabine (ECX) [7]. Those trials established the benefit of pre-operative chemotherapy in patients with locally advanced gastric cancer. However, given the heterogeneity of gastric cancer and the variable response to preoperative therapies, it is crucial to accurately identify those patients who respond to neoadjuvant chemotherapy and thus benefit from it, and those patients who do not respond, allowing for reassessment of their treatment options. 

## 3. Gastric Cancer Staging and Tumor Response Grade

The 8th edition of the AJCC cancer staging manual, published in 2017, introduced an updated staging system for gastric cancer [8]. Due to the lack of a validated clinical staging system (cTNM) for gastric cancer in prior editions, the pathologic staging system (pTNM) was used instead by treating physicians in the pre-surgical setting, without complete surgical pathology information. However, this created confusion and lacked accurate prognostication. The new AJCC staging system filled this gap by introducing a clinical staging system [9]. Additionally, a new post-neoadjuvant TNM system was introduced (labeled as ypTNM), which takes into consideration the use of neoadjuvant therapies and pathologic response. Stage is based on identification of the deepest focus of residual viable cancer cells within the gastric wall for the ypT-stage, and the presence of malignant cells in regional lymph nodes for the ypN-stage. Kim et al. analyzed approximately 9000 patients with clinical stage 1–4 gastric cancer who underwent neoadjuvant therapy followed by resection. In their study, post-neoadjuvant stage (ypStage) was able to more accurately predict survival compared to clinical staging. Patients who were downstaged post-operatively had a statistically significant improvement in OS. For example, patients with clinical stage 3 disease who were downstaged to pathologic stage 1 or 2 had 5-year OS rates of 54.6% and 33.1% compared to 19.1% if the stage remained the same [10].

According to the sixth edition of the Japanese Gastric Cancer Treatment Guidelines published in July 2021, tumor response to chemotherapy should be evaluated by the Japanese Classification of Gastric Carcinoma or the Response Evaluation Criteria in Solid Tumors (RECIST) [11]. RECIST 1.1 has become the gold standard for the assessment of treatment response in solid tumors [12]. It provides a set of rules and radiographic criteria that need to be met in order to characterize response to treatment as complete response, partial response, progressive disease and stable disease. Another way to assess tumor response to treatment is by assessing the tumor response grade (TRG). Tumor response grade or tumor regression score, defined as a measurable histologic response of tumor cells to neoadjuvant therapy, has been used a prognostic marker in several malignancies, including breast [13], pancreatic [14] and colorectal carcinoma [15,16]. Complete response, or 100% treatment response, refers to a lack of viable tumor cells and the presence of fibrosis or fibroinflammation within the lesion. No response, or 0% treatment response, indicates a lack of any type of evident treatment effect. Incomplete response refers to the presence of residual cancer cells along with some degree of treatment effect [17]. While RECIST can provide valuable information in the pre-operative setting, TRG can only be measured in the post-operative setting, as it requires histopathologic examination of the resected surgical specimen. 

## 4. Tumor Response Grading Systems

TRG is a histopathological grading system that evaluates the degree of response of tumor cells to preoperative therapy. Many different grading systems have been developed to document TRG, such as those by Becker et al. [18], Mandard et al. [19], Ryan et al. [20], Japanese Gastric Cancer Association (JGCA) [11,21], Martin-Romano et al. [22], Tsekrekos et al. [23,24] (Table 1). Each TRG uses a different scaling system and interpretation guidelines, rendering it difficult to achieve a direct comparison of results between different systems.

**Figure 1:**
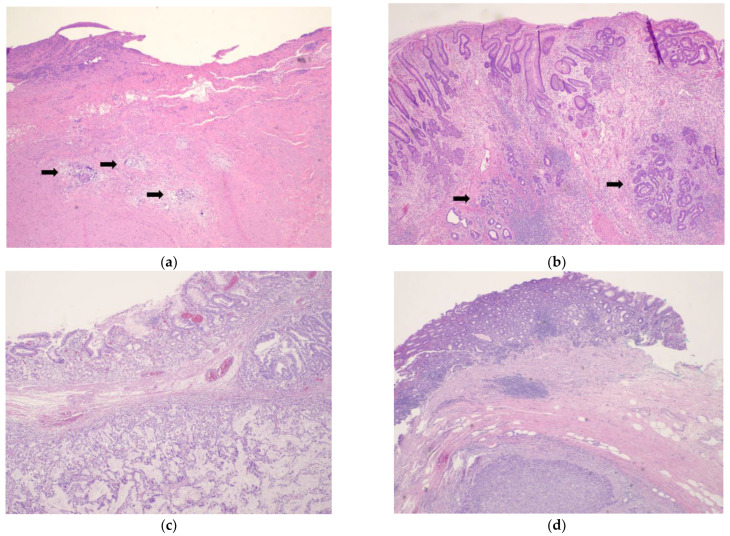
Representative histologic images of tumor regression grades assigned using schema in CAP Protocol Version 4.1.0.0, (hematoxylin–eosin staining) (**a**) Rare small groups of carcinoma cells (→) are present within a fibrotic tumor bed in an esophagogastrectomy specimen, corresponding to TRG 1; (**b**) residual foci of adenocarcinoma (→) persist in a gastrectomy that showed evident treatment effect in the form of ulcer, necrosis, inflammation, and calcifications, consistent with TRG 2; (**c**,**d**) extensive residual tumor is readily apparent, with no evidence of tumor regression, corresponding to TRG 3.

Assessing TRG can be challenging, and it can produce variable results between pathologists. The National Comprehensive Cancer Network (NCCN) recommends using the grading system currently used by the College of American Pathologists (CAP), a modification of the system developed by Ryan et al. [3,20,25]. The modification made to the Ryan et al. system is defining two separate grades for complete (no residual tumor) and near complete response (single cells or rare small groups of tumor cells) as TRG 0 and TRG 1, respectively. NCCN additionally specifies that TRG 0 is reserved for patients with complete response of the primary tumor, including lymph nodes. [3] The CAP Protocol for reporting gastric carcinomas suggests using this modified system (Table 1, Figure 1), given its good interobserver reproducibility. However, use of other systems for assessment of treatment response, including estimation of response as the percentage of the gross lesion comprised of viable tumor cells in relation to fibrosis, is not precluded by the Protocol [3,20,25,26]. In 2016, Martin-Romano et al. proposed the use of a four-tier grading system to assess treatment response in the lymph nodes [22]. In 2019, Tsekrekos et al. published the results of a Delphi study, in which the authors proposed a new staging system, a modification of the Becker et al. TRG system, along with a three-tier grading system to evaluate nodal response. In their system, lymph node TRG was defined as grade a (complete regression), grade b (partial regression) and grade c (no regression) [24]. Similarly, another Delphi study published in 2021 proposed an identical TRG system to the one proposed by Tsekrekos et al., for evaluation of treatment response in esophageal and EGJ malignancies [27].

A study assessing interobserver reproducibility of different TRG systems measured the kappa (κ) statistic (with higher κ score indicating higher reproducibility), and found that the TRG systems by Mandard, Becker and JGCA have κ scores of 0.44, 0.52 and 0.28, respectively [28]. Ryan et al. compared a three-point and five-point TRG system in 60 patients with locally advanced rectal cancer who underwent neoadjuvant therapy and calculated κ scores of 0.84 and 0.64, respectively [20]. In 2021, Tsekrekos et al. calculated Kendall’s coefficient of concordance (W) for their proposed TRG system, which is a value that measures the strength of agreement between multiple ratings, and found a W score of 0.70 (good agreement) for assessment of the primary tumor and 0.52 (moderate agreement) regarding lymph node assessment. When stratified by level of experience between pathologists, the W score for lymph node assessment reached 0.64 for subspecialized pathologists vs. 0.45 for nonspecialized. There was no significant difference in W score for primary tumor assessment [23]. However, as TRG can only be evaluated only after surgical resection, its role in clinical practice remains ambiguous.

## 5. Tumor Response Grade Prognostication

Several studies have demonstrated an association of TRG with OS. In 1999, Lowy et al. published the results of three randomized controlled trials that investigated neoadjuvant treatment of patients with gastric cancer. In their study, they analyzed 83 patients with ≥T2, M0 gastric cancer who received preoperative chemotherapy followed by resection. Sixty-one patients underwent R0 resection, with sixteen of them having a pathologic response; three patients had a complete histologic response, ten had a partial response (defined as <10% of viable tumor cells) and three had a minor response (10–50% viable tumor cells). In their multivariate analysis, tumor response to chemotherapy was the only factor associated with overall survival, with a relative risk of 0.44 [29]. In a study published by Becker et al. in 2003, 36 patients with locally advanced gastric cancer (LAGC) received neoadjuvant etoposide, doxorubicin and cisplatin followed by resection. No patients had a complete histologic response to therapy. Only four patients had <10% of residual disease, whereas 23 patients had >50% residual tumor. Univariate analysis demonstrated a statistically significant association of tumor regression with survival [18]. In another study published in 2011, the investigators evaluated TRG scores in 480 patients with LAGC who received neoadjuvant platinum-based chemotherapy. The study showed 21.2% of patients had complete (3.3%) or subtotal tumor regression (defined as <10% residual cancer, 17.9%), whereas 25.2% had partial tumor regression (10–50% residual cancer). Multivariate analysis revealed that tumor regression and post-operative lymph node status were factors associated with survival. Patients with complete/subtotal tumor regression had a mean survival of 128.6 months versus 61.9 months for partial tumor regression [30]. In another study by Xie et al. published in 2021, 249 patients with >T2 and/or N1 disease underwent oxaliplatin-based (69%) or non-oxaliplatin-based neoadjuvant therapy. Using the CAP TRG system, patients with TRG 0–1 had 3-year and 5-year survival rates of 85.2% and 74.5%, respectively, compared to 56.1% and 44.1% in patients with TRG of 2. Patients with TRG 3 had significantly worse survival rates with 3-year and 5-year rates of 28.2% and 23.0%, respectively. Multivariate analysis showed that TRG and margin status were factors associated with survival, whereas TRG was the only factor associated with recurrence-free survival [31]. Sinnamon et al. evaluated 117 patients with clinical T2+ and/or N+ gastric cancer who underwent preoperative chemotherapy followed by resection. Amongst the most commonly used chemotherapy regimens, 41% of patients received ECF/ECX, 26% received folinic acid, fluorouracil and oxaliplatin (FOLFOX), and 11.1% received FLOT. Using the CAP TRG, the majority of patients were found to have no response to treatment (TRG 3, 58%). TRG 1 and TRG 2 were found in 15% and 21%, respectively, whereas complete response (TRG 0) was only found in 5.1% of the cohort. The increasing TRG score showed a statistically significant association with decreasing OS (HR 1.49, *p* = 0.026). Specifically, patients with TRG 0, 1, 2 and 3 had survival rates of 86.9, 74.5, 51.5 and 27 months, respectively. No association between TRG and choice of chemotherapeutic regimen was observed. Post-therapy pathologic lymph node status was also associated with survival (HR 1.93, *p* = 0.026) [28]. In 2020, Reim et al. investigated the impact of post-neoadjuvant chemotherapy nodal status in patients with GEJ (Siewert type 2 and 3) and gastric adenocarcinoma. Patients with no evidence of lymph node disease nor regression had the best prognosis compared to lymph node positive/regression negative patients, who had the worst prognosis (HR 1.496, 95% CI 1.35–1.65, *p* ≤ 0.001). Patients with lymph node positive disease and evidence of regression had a statistically significant improvement in prognosis compared to patients with positive lymph nodes and no regression (*p* = 0.01). Univariate analysis demonstrated that TRG grade of the primary tumor, among other factors, was associated with survival. Multivariate analysis showed that lymph node disease/regression status was associated with prognosis (HR 1.23, 95% CI 1.1–1.38, *p* < 0.001) [32]. A retrospective study published in 2016 evaluated TRG scores of the primary tumor and lymph nodes in patients with cT3-T4 and/or N+, M0 gastric adenocarcinoma who underwent either perioperative chemotherapy or induction chemotherapy followed by chemoradiation. The authors defined Becker grades Ia and Ib as a major pathologic response. A four-tier system was used to define nodal response; grade A for patients with true negative lymph nodes and no signs of treatment effect, grade B for positive lymph nodes with no treatment effect, grade C for positive lymph nodes with treatment effect, and grade D for negative lymph nodes with treatment effect. Thirty-four patients received perioperative chemotherapy and forty-six received chemoradiation. There was a statistically significant difference in major pathologic response between the two treatment groups (32% vs. 58%, respectively, *p* = 0.001). Additionally, more patients in the chemoradiation group were found to have grade D lymph node response and major pathologic response of the primary tumor compared to the chemotherapy group (23% vs. 3%, *p* = 0.019). There was no difference in the number of patients with positive lymph nodes, as well as in the number of patients who were downstaged. While there was no significant difference in OS between the two treatment groups, a subgroup analysis showed that patients with grade D lymph node response had improved progression-free survival (PFS) compared to patients with grade B/C response (79% vs. 31%) with HR 3.96 (95% CI 1.78–13.32, *p* = 0.043). There was no difference in 5-year OS and PFS between patients with grade A and D lymph node response, rendering the survival of patients with completely treated lymph node disease equal to those with N0 disease prior to the initiation of preoperative therapies [22]. Such improvements in survival highlight the impact of tumor response to neoadjuvant therapy among “responders” and “non responders”. 

While the studies above show an association of TRG with OS, other studies challenge this. In 2007, Mansour et al. analyzed the histologic response of 168 patients with gastric adenocarcinoma who underwent neoadjuvant chemotherapy followed by resection. Even though the chemotherapeutic regimens varied among the cohort, the majority of patients (68%) received cisplatin-based chemotherapy. Eighteen patients (11%) had no evidence of histologic response and only two patients had a complete response. The majority of patients had <20% histologic response, and 23% had a response >50%. In univariate analysis, histologic response ≥50% was associated with 3-year disease-specific survival of 69% versus 44% in patients with <50% response. In multivariate analysis, however, lymph node involvement and perineural or vascular invasion were found to be associated with disease-specific survival, but the effect of histologic response (scored as above or below 50%) was not significant. Notably, Cisplatin-based chemotherapy regimens were three times more likely to produce a histologic response >50% [26]. In 2016, Smyth et al. evaluated the association of TRG and OS in patients who were enrolled in the MAGIC trial. Univariate analysis demonstrated an association between TRG scores and lymph node involvement on OS. However, multivariate analysis showed that lymph node involvement, and not tumor regression, was predictive of survival [5,33].

## 6. Radiographic and Endoscopic Response to Chemotherapy and Prognostication

As mentioned earlier, the response of solid tumors to chemotherapy can be evaluated in the preoperative setting according to RECIST 1.1 criteria by identifying a target lesion with measurable disease. Gastric cancer patients with radiographic response to neoadjuvant therapy have been shown to have improved disease-free and OS compared to non-responders [34,35]. However, the location and volume of a gastric malignancy cannot be reliably and consistently identified and measured on computed tomography, rendering the use of RECIST criteria for primary gastric lesions challenging. For this reason, alternate measurable lesions are pursued (lymph nodes, liver metastasis) [12]. Additionally, radiographic assessment can underestimate tumor response to chemotherapy, as it cannot distinguish chemotherapy-induced inflammatory response and fibrosis from remaining tumor [34]. Chemotherapy-related changes result in distortion of the layers of the gastric wall, which in turn causes evaluation of tumor response with endoscopic ultrasound (EUS) to correlate poorly with the histologic response seen on surgical specimens [36]. In 1999, Ajani et al. evaluated the accuracy of presurgical EUS and laparoscopy staging in patients with LAGC. In their study, 13 patients with LAGC underwent staging with EUS after completion of neoadjuvant therapy and before surgical intervention. EUS was able to accurately predict pathologic staging in only 3 out of 13 patients (13%), whereas 8 patients were understaged [37]. In another study, 40 patients with LAGC received neoadjuvant therapy and were restaged with EUS and CT before undergoing gastrectomy. EUS was found to be 47% and 39% accurate in correctly determining the pathologic T and N stage, respectively, whereas computed tomography was 57% and 37% accurate. EUS downstaging of T and/or N status was significantly associated with OS (HR 0.12, 95% CI 0.01–0.91 *p* = 0.04), whereas CT downstaging did not reach statistical significance [38]. 

The use of positron emission tomography (PET) CT has been investigated as a pre-operative modality in an attempt to guide preoperative treatment and improve prognostication. In 2021, Goodman et al. published the results of the Cancer and Leukemia Group B (CALGB) 80803 phase II randomized trial (Alliance). The study analyzed 225 patients with esophageal (40.6%) or esophagogastric junction (59.4%) adenocarcinomas (Siewert 1 and 2) with ≥T2 and or N+, M0 disease who received preoperative chemotherapy, followed by chemoradiation. PET response was defined as ≥35% decrease (responders) or <35% decrease (non-responders) in tumor glucose standard uptake values (SUV) compared to pre-treatment PET SUV and was assessed 36 to 42 days following induction chemotherapy with either FOLFOX or carboplatin–paxlitaxel (CP). The authors reported that pathologic complete response (pCR) rates (defined as no viable tumor in the specimen, including lymph nodes; ypT0N0) of the two chemotherapy regimens in PET-responders were 40.3% and 14.1% for FOLFOX and CP, respectively, with corresponding 5-year OS rates of 53% and 43.9%. Patients who were deemed non-responders crossed over to the alternate chemotherapy regimen and achieved pCR of 20% if switched to FOLFOX from CP, and 18% if switched to CP from FOLFOX, with 5-year OS rates of 40.4% and 37.5%, respectively. Interestingly, the difference in 5-year OS between the two groups did not reach statistical significance (HR 1.34, 0.94–1.92, *p* = 0.1065). Patients who received induction FOLFOX had a higher rate of pathologically node-negative disease compared to CP, with rates of 84.1% vs. 66.7% for PET-responders and 71.4% vs. 59% for non-responders, respectively (*p* = 0.035) [39]. In 2007, the results of the “Metabolic response evaluation for individualisation of neoadjuvant chemotherapy in oesophageal and oesophagogastric adenocarcinoma” (MUNICON) trial were made available. The authors found no significant difference between the choice of chemotherapeutic regimen and the PET response in patients with Siewert type 1 or 2 EGJ adenocarcinoma who underwent neoadjuvant chemotherapy. PET responders were found to have a higher baseline SUV compared to non-responders (8.3 vs. 6.8, *p* = 0.018) and a higher rate of R0 resection (96% vs. 74%, *p* = 0.002). While 58% of PET-responders demonstrated a major histologic response, no histologic response was observed in PET non-responders. Patients with both a PET response and a complete or subtotal histologic response had improved OS compared to PET responders without a histologic response (HR 4.55, *p* = 0.004). There was no difference in survival between PET responders without histologic response and PET non-responders [40].

The use of PET CT in gastric cancer in assessing the response to preoperative therapies is under investigation. A study from Italy published in 2017 showed that in patients with resectable T3–T4 and/or N+, M0 gastric or EGJ adenocarcinoma undergoing perioperative chemotherapy with irinotecan–oxaliplatin–capecitabine, pathologic response correlated with the baseline SUV level of the primary tumor and not with the SUV change following treatment [41]. In another study from Germany published in 2010, the authors did not identify a statistically significant association between pre- and post-treatment SUV levels and histologic response or prognosis in patients with LAGC undergoing neoadjuvant treatment with cisplatin–leucovorin-5FU (PLF) [42]. 

There is evidence that histologic subtype and tumor location play a major role when evaluating gastric cancer patients with PET. Ott et al. analyzed 71 patients with LAGC who underwent preoperative therapy with two cycles of PLF, followed by resection. One-third (22 patients, 30.9%) did not demonstrate pre-operative FDG-avidity. Sixty-eight percent of those patients were found to have non-intestinal type gastric cancer (12 patients with signet ring cells) compared to 47% in patients with FDG-avid tumors (23 out of 49 patients, 11 with signet ring cells). The presence of signet ring cells was statistically associated with non-evaluable disease by PET (*p* = 0.013), likely related to the presence of mucin and decreased FGD concentration within the tumor, as well as decreased uptake of glucose due to the lack of expression of glucose transporter-1 on the tumor cell membrane. Notably, there was a significant difference in the rate of FDG-non avid and FDG-avid tumors located in the distal two thirds of the stomach (45.4% vs. 22.4%, *p* = 0.029) [43]. 

## 7. Future Directions

Undoubtedly, response to preoperative chemotherapy is associated with a favorable tumor biology and indicates tumor chemosensitivity. Conversely, nonresponse to chemotherapy points to a biologically aggressive and chemotherapy-resistant tumor. Correctly identifying the group of patients in whom chemotherapy does not have an effect in the neoadjuvant setting will allow for potential alteration of the chemotherapeutic regimen and provide a “second chance” for disease control and ultimately, cure. Randomized controlled trials are needed to better understand the value of radiographic and histologic assessment of tumor response in prognostication and disease management. Future studies will need to focus on identifying the ideal preoperative methods and biomarkers that can accurately predict and assess tumor response. Additionally, while newer TRG systems incorporate the tumor response of metastatic lymph nodes in preoperative therapies, this is inconsistently reported in studies and will need to be investigated further as a possible prognostic factor. A widely accepted post-treatment histologic grading system is needed, as it can be a valuable tool in comparing study results and treatment responses in the future. Advances in radiomics and genomics will improve clinical decision making in gastric cancer treatment and advance patient care. 

## 8. Conclusions

There is evidence that tumor response grade following neoadjuvant therapy for gastric cancer is associated with survival. Although multiple grading systems exist, there is need for a single, unified, and widely accepted grading system that would allow for direct comparison of TRG results between studies. This would allow clinicians to provide complete counseling and supply their patients with more accurate prognostication.

## Figures and Tables

**Table 1:** Tumor response grading systems.

TRG System	Grade—Interpretation
Mandard et al. (1994) [19]	1—No residual tumor, presence of fibrosis 2—Rare residual tumor cells scattered through fibrosis 3—Increased residual tumor cells but fibrosis is predominant 4—Residual tumor cells outgrowing fibrosis 5—Absence of regression
Becker et al. (2003) [18]	1a—Complete response; no residual tumor per tumor bed 1b—Subtotal response; <10% residual tumor per tumor bed 2—Partial response; 10–50% residual tumor per tumor bed 3—Minimal or no response; >50% residual tumor per tumor bed
Ryan et al. (2005) [20]	1—No viable tumor cells or single cells or small groups of tumor cells 2—Residual tumor outgrown by fibrosis 3—No fibrosis with extensive residual tumor or significant fibrosis outgrown by tumor
NCCN-CAP (Modified Ryan et al.) [3,25]	0—Complete response; no viable tumor cells, including lymph nodes 1—Near complete response; single cells or rare small groups of tumor cells 2—Partial response; residual tumor with evident regression but more than single cells or rare small groups of tumor cells 3—Poor or no response; no evidence of tumor regression
JGCA (2011) [11]	0—No effect seen 1a—Very slight effect; More than 2/3 of viable tumor present 1b—Slight effect; Between 1/3 and 2/3 of viable tumor 2—Considerable effect; Less than 1/3 of viable tumor 3—Complete response; no tumor cells seen
Martin-Romano et al. (2016) [22]	Primary tumor Use Becker TRG Lymph nodes A—Negative lymph nodes with no evidence of treatment effect (true negative) B—Positive lymph nodes with no evidence of treatment effect C—Positive lymph nodes with evidence of tumor regression/treatment effect D—Negative lymph nodes with complete pathologic response
Tsekrekos et al. (Modified Becker et al.) 2019 [24]	Primary tumor 1—Complete response; no residual tumor cells, only signs of regression 2—Less than 10% residual tumor cells 3—Between 10–50% residual tumor cells 4—More than 50% residual tumor cells Lymph nodes a—Complete regression; all lymph nodes are negative, with signs of regression b—Partial regression; lymph nodes with tumor cells and signs of regression c—No regression; lymph nodes with tumor cells and no signs of regression

## Data Availability

Data sharing not applicable.
